# Impact of Nitric Oxide (NO) on the ROS Metabolism of Peroxisomes

**DOI:** 10.3390/plants8020037

**Published:** 2019-02-10

**Authors:** Francisco J. Corpas, Luis A. del Río, José M. Palma

**Affiliations:** Group of Antioxidants, Free Radicals and Nitric Oxide in Biotechnology, Food and Agriculture, Department of Biochemistry and Cell and Molecular Biology of Plants, Estación Experimental del Zaidín, Consejo Superior de Investigaciones Científicas (CSIC), Profesor Albareda 1, 18008 Granada, Spain; luisalfonso.delrio@eez.csic.es (L.A.d.R.); josemanuel.palma@eez.csic.es (J.M.P.)

**Keywords:** catalase, monodehydroascorbate reductase, tyrosine nitration, nitric oxide, peroxisome, reactive oxygen species, *S*-nitrosation, superoxide dismutase

## Abstract

Nitric oxide (NO) is a gaseous free radical endogenously generated in plant cells. Peroxisomes are cell organelles characterized by an active metabolism of reactive oxygen species (ROS) and are also one of the main cellular sites of NO production in higher plants. In this mini-review, an updated and comprehensive overview is presented of the evidence available demonstrating that plant peroxisomes have the capacity to generate NO, and how this molecule and its derived products, peroxynitrite (ONOO^−^) and *S*-nitrosoglutathione (GSNO), can modulate the ROS metabolism of peroxisomes, mainly throughout protein posttranslational modifications (PTMs), including *S*-nitrosation and tyrosine nitration. Several peroxisomal antioxidant enzymes, such as catalase (CAT), copper-zinc superoxide dismutase (CuZnSOD), and monodehydroascorbate reductase (MDAR), have been demonstrated to be targets of NO-mediated PTMs. Accordingly, plant peroxisomes can be considered as a good example of the interconnection existing between ROS and reactive nitrogen species (RNS), where NO exerts a regulatory function of ROS metabolism acting upstream of H_2_O_2_.

## 1. Introduction

Peroxisomes are organelles with an essential oxidative metabolism present in almost all categories of eukaryotic cells. In higher plants, these organelles are recognized to have a versatile metabolism because their enzymatic composition can adapt to different cell and organ types, stages of development, and environmental conditions [1,2,3,4,5,6]. However, there is a common battery of enzymes that are present in all types of plant peroxisomes. This includes a set of antioxidant systems whose functions are to keep under control the internal active metabolism of reactive oxygen species (ROS), mainly superoxide radicals (O_2_^·−^) and hydrogen peroxide (H_2_O_2_). These ROS are generated under physiological conditions by different pathways, such as purine catabolism, fatty acid β-oxidation, and photorespiration [7,8,9,10]. These antioxidant systems acquire a special relevance in those situations where the ROS generation is intensified, like under plant stress conditions [11]. 

In recent years, different experimental data have demonstrated that plant peroxisomes also have the capacity to generate another free radical—nitric oxide (NO)—and a family of derived molecules designated as reactive nitrogen species (RNS), including peroxynitrite (ONOO^−^) [12] and *S*-nitrosoglutathione (GSNO) [13]. The production of these two families of reactive species—ROS and RNS—raises new questions about their potential functions in peroxisomes, either as simple byproducts of the peroxisomal metabolism or perhaps having a regulatory function in the peroxisome and also outside these organelles, due to the characteristic signaling properties of ROS and RNS.

In this work, the interconnections existing between the metabolism of ROS and RNS in peroxisomes are presented. In this relationship, NO exerts a regulatory function by controlling the activity of some target enzymes through posttranslational modifications (PTMs), mainly *S*-nitrosation (or *S*-nitrosylation) and tyrosine nitration. It should be pointed out that the NO-generating capacity of peroxisomes may have significant implications in the cellular metabolism of plants under physiological conditions, including leaf senescence [14], pollen tube growth [15], and auxin-induced root organogenesis [16]. However, peroxisomal NO metabolism is particularly exacerbated under oxidative stress situations induced by abiotic conditions like salinity [17], and the heavy-metals cadmium [12,18], and lead [19].

## 2. Nitric Oxide Generation in Plant Peroxisomes

In higher plants, NO is a key signaling molecule [20,21] involved in numerous processes, including seed germination [22,23], primary and lateral root growth [24,25], plant development [26,27], stomatal closure [28], flowering [29], reproductive tissues [15,30,31], fruit ripening [32,33], senescence [14,34], abiotic stresses [35,36,37,38,39] and biotic stresses [40]. However, the enzymatic source(s) of NO in plant cells is still a controversial matter subject to intense discussions [41,42,43]. Different pieces of biochemical evidence have demonstrated the presence of L-arginine-dependent nitric oxide synthase (NOS)-like activity in plant peroxisomes. Data accumulated during the last twenty years indicate that the hypothetical protein responsible for the NO generation in peroxisomes has biochemical requirements similar to that of animal NOS, including substrate, cofactors and sensitivity to inhibitors [14,44], dependence on calcium and calmodulin [45], as well as dependence on the mechanism of the import system to peroxisomes through a peroxisomal targeting signal type 2 (PTS-2) [46]. The known biochemical properties of the protein responsible for NO generation in plant peroxisomes, in comparison with those described for animal NOS, are summarized in Table 1. Additionally, there are experimental data that have corroborated the presence of NO in plant peroxisomes and that were obtained by complementary approaches, including electron paramagnetic resonance (EPR) spectroscopy, ozone chemiluminescence, and NO-specific fluorescence probes [14,19]. It should be mentioned that in other cellular compartments a reductive NO generation involving nitrite/nitrate or nitrate reductase (NR) has been described, as well as a non-enzymatic production of NO at acidic pH in the presence of reductants like ascorbate [43,47]. However, peroxisomes have at oxidative metabolism and, to our knowledge, there is not any experimental evidence of the presence of nitrite/nitrate or NR in these plant organelles. Moreover, it has been reported that peroxisomes have an alkaline pH [48], what suggests that the mentioned non-enzymatic generation of NO in peroxisomes is not likely under normal physiological conditions.

Similarly, in animal peroxisomes, the presence of an inducible NOS isozyme [49,50], which is imported to the peroxisomal matrix using a PTS2 [51], has also been demonstrated. In conclusion, the above data indicated for the protein responsible for NO generation in peroxisomes from plant origin are in good agreement with the data reported for the animal peroxisomal NOS activity.

## 3. Peroxisomal Proteins: Targets of NO-mediated PTMs

At present, the number of potential targets that undergo NO-mediated PTMs is increasing. This is due to the identifications obtained by specific proteomic methodologies combined with biochemical analyses, such as the biotin switch method and labeling with isotope-coded affinity tags (ICAT). These approaches have also allowed confirming whether a specific protein is *S*-nitrosated and/or nitrated. In some cases, even the affected amino acid residues of the protein have been identified [52]. Furthermore, the existence of any NO-derived PTM is additional evidence of, at least, the presence of NO and its derived molecules in a specific subcellular compartment [53]. So far, the number of identified plant peroxisomal proteins susceptible to undergo a specific NO-derived PTM has also increased with the development of the mentioned methodologies. The characteristic peroxisomal proteins that have been identified as targets of NO in higher plants are summarized in Table 2. Among the different peroxisomal proteins undergoing NO-derived PTMs, in this article, we have focused on some of the key antioxidant enzymes of peroxisomes, including catalase (CAT), monodehydroascorbate reductase (MDAR), and copper-zinc superoxide dismutase (CuZnSOD).

### 3.1. Catalase (CAT, EC 1.11.1.6)

CAT is a heme-containing protein and one of the key H_2_O_2_-scavenging enzymes present in prokaryotic and eukaryotic cells [64,65,66,67]. Additionally, CAT is recognized as a constitutive enzyme of all kinds of peroxisomes from eukaryotic cells, being used as a biochemical marker of these organelles. The information available, at present, indicates that this enzyme is the main target of NO in animals and plants. In fact, initial in vitro assays showed that the bovine liver CAT was rapidly and reversibly inhibited by NO [68,69]. In plants, using purified tobacco CAT, similar studies demonstrated that both NO donors and ONOO^−^ (a nitrating molecule) had the capacity to inhibit the enzyme activity [70]. More recently, studies carried out in different plant species have shown that CAT is a target of *S*-nitrosation in sunflower hypocotyls [60], pea leaves [55], and Arabidopsis [56], and of tyrosine nitration in pepper fruits [61]. Moreover, it was demonstrated that both *S*-nitrosation and tyrosine nitration inhibited CAT activity in pea leaves and pepper fruits [55,61]. It has been proposed that the potential target of *S*-nitrosation in Arabidopsis CAT is Cys86 [56], although this should be corroborated by specific mass spectrometry analyses. However, it must be taken into account that NO could also interact with the Fe atoms present in the heme groups of CAT, forming a metal nitrosyl complex, that perhaps could affect its activity, although, to our knowledge, there is no information on this mechanism in plant CAT. In any case, all the data available suggest that NO acts upstream of H_2_O_2_, thereby regulating CAT activity. This inhibition of CAT by NO could imply a lower capacity to remove H_2_O_2_, and consequently it could be well correlated with those physiological or adverse processes that have associated an increase of their oxidative metabolism [18,61].

### 3.2. Monodehydroascorbate Reductase (MDAR, EC 1.6.5.4) 

This enzyme is part of the ascorbate-glutathione (ASC-GSH) cycle, whose function is also to control the cellular content of H_2_O_2_ [71]. The ASC-GSH cycle is present in different subcellular compartments, including peroxisomes [72,73,74]. However, very little information is available on how RNS can regulate the specific isozymes of this cycle present in peroxisomes. MDAR catalyzes the NADH-dependent conversion of monodehydroascorbate to ascorbate, and peroxisomal MDAR has been characterized in pea leaves [75] and Arabidopsis [76]. Further in vitro analysis of recombinant MDAR from pea leaf peroxisomes in the presence of nitrating or *S*-nitrosylating agents (ONOO^−^ or GSNO, respectively) demonstrated that both processes caused inhibition of the MDAR activity [63]. Mass spectrometric analysis and site-directed mutagenesis confirmed that Tyr345 was the primary site of nitration by ONOO^−^ responsible for the inhibition of MDAR activity. On the other hand, in silico analysis of the MDAR indicated that Cys68 was the best candidate for *S*-nitrosylation [63]. This implies a possible modulation in peroxisomes of the ascorbate regeneration and the H_2_O_2_ scavenging by RNS.

### 3.3. Superoxide Dismutase (SOD; EC 1.15.1.1) 

Superoxide dismutases (SODs) are a family of metalloenzymes that catalyze the disproportionation of O_2_^·−^ radicals into H_2_O_2_ and O_2_. In higher plants, there are three main types of SODs, containing prosthetic metals Mn (Mn-SODs), Fe (Fe-SODs), or Cu plus Zn (Cu,Zn-SODs) [77,78]. The presence of SOD activity in peroxisomes was reported for the first time in plant tissues—in pea (*Pisum sativum* L.) leaves—in the early 1980s [79]. However, this report, in general, passed unnoticed and was even questioned until it was described in human cells years later [80]. Since then, the occurrence of different types of SODs in plant peroxisomes has been described in at least ten distinct plant species [11,78]. At present, SOD is considered a constitutive enzyme in all types of peroxisomes, although the family of isozyme present depends on the organ and plant species. 

In relation to the susceptibility of SOD to different RNS-induced modifications, previous reports indicated that the recombinant human Mn-SOD and Cu,Zn-SOD were prone to be inactivated by ONOO^−^ [81,82]. In the case of plant peroxisomes, recently the recombinant peroxisomal Cu,Zn-SOD (designated as CSD3) was obtained in Arabidopsis, and in vitro assays in the presence of nitrating or *S*-nitrosylating agents showed that 500 μM ONOO^−^ provoked a 65% inhibition of the Cu,Zn-SOD activity, whereas GSNO did not cause any effect [62]. Regarding mass spectrometric analyses, Tyr115 was identified as the potential target of nitration [62]. Accordingly, SOD seems to be a relevant protein to be further investigated as a target of NO-mediated PTMs, since it appears to be sensitive to exert some discrimination between nitration and nitrosation processes.

## 4. Conclusions and Future Perspectives 

**Figure 1:**
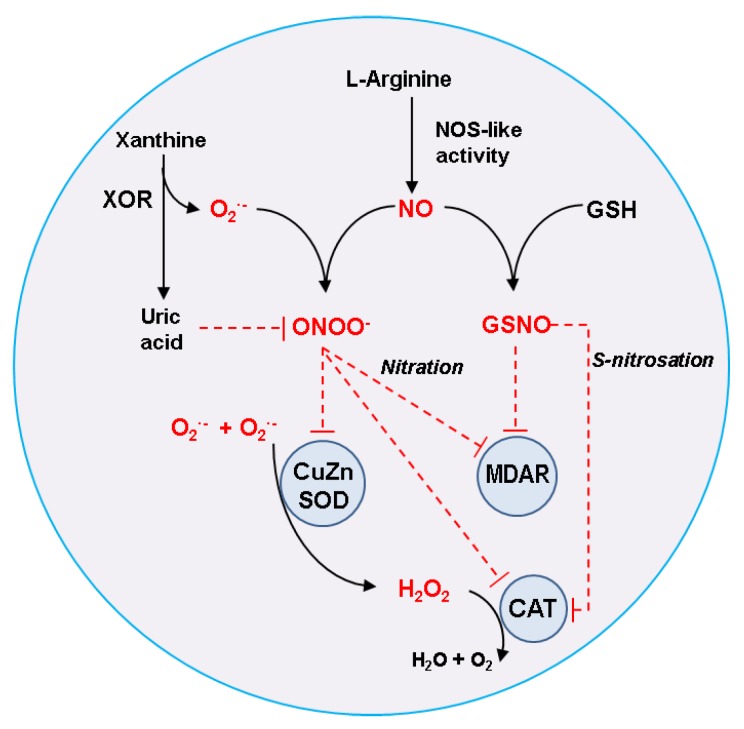
**The interrelationship between nitric oxide (NO) metabolism and antioxidant enzymes in plant peroxisomes.** Peroxisomal xanthine oxidoreductase (XOR) activity produces uric acid and superoxide radicals (O_2_^·−^). On the other hand, an L-arginine-dependent nitric oxide synthase (NOS)-like activity generates NO, which can react with O_2_^·−^ to give rise to peroxynitrite (ONOO^−^), which is a powerful oxidant and strong nitrating molecule that can mediate posttranslational modifications (PTMs), such as tyrosine nitration. NO can also interact with reduced glutathione (GSH) to form *S*-nitrosoglutathione (GSNO), a NO donor that can mediate S-nitrosation reactions. Uric acid is a recognized ONOO^−^ scavenger that could be part of a mechanism of peroxisomal auto-regulation. With all these components, the identified targets of NO-derived PTMs in peroxisomes, catalase (CAT), copper, zinc superoxide dismutase (CuZnSOD), and monodehydroascorbate reductase (MDAR) can undergo inhibition of their activity either by nitration or *S*-nitrosation.

Plant peroxisomes have relevant antioxidant systems comprised mainly of CAT, SOD, and the ASC-GSH cycle, which are present in all types of plant peroxisomes [7]. Likewise, results obtained in previous research works have demonstrated that besides an active ROS metabolism in peroxisomes, these organelles also have an active RNS metabolism. Although there are few specific studies on how distinct RNS can regulate the different peroxisomal antioxidant systems, the data available suggest the NO may act upstream of the H_2_O_2_ metabolism. A scheme based on previous reports [7,11,83,84], showing how NO can modulate the activity of peroxisomal antioxidant enzymes throughout either nitration or *S*-nitrosation, is presented in Figure 1. The peroxisomal xanthine oxidoreductase (XOR) activity catalyzes the oxidation of xanthine with the production of uric acid and O_2_^·−^ [85]. On the other hand, L-arginine-dependent NOS-like activity generates NO, which can react with O_2_^·−^ to produce ONOO^−^, a powerful oxidant and strong nitrating molecule that can mediate PTMs through tyrosine nitration [86]. NO can also interact with reduced glutathione (GSH) to form GSNO, a NO donor that can mediate *S*-nitrosation of proteins [87]. Uric acid is a recognized inhibitor of ONOO^−^-mediated toxicity [88,89], and this brings out a new potential mechanism of peroxisomal auto-regulation through this powerful nitrating molecule. In this scenario, the identified targets of NO-derived PTMs in peroxisomes, CAT, CuZnSOD, and MDAR, which are either directly or indirectly linked to the H_2_O_2_ pool, are key points to be modulated by nitration or *S*-nitrosation. 

In summary, the data presently available indicate that plant peroxisomes contain multiple elements of ROS and RNS metabolism, where NO seems to act upstream of H_2_O_2_ routes throughout the regulation of the peroxisomal antioxidant enzymes. Nevertheless, it should be taken into account that both NO and H_2_O_2_ could be released to the cytosol, acting as signal molecules among the different subcellular compartments. However, in plants under certain abiotic stress conditions an overproduction of H_2_O_2_ and NO could take place in peroxisomes, and a high accumulation of these signal molecules can mediate a nitro-oxidative stress in plant cells [11,90]. 

## Figures and Tables

**Table 1:** Biochemical requirements of the peroxisomal protein responsible for the L-arginine-dependent nitric oxide synthase (NOS)-like activity in higher plants.

Requirements	Peroxisomal NOS-Like Protein
Substrate	L-Arginine
Cofactor requirement	NADPH, Ca^2+^, FAD, FMN, BH_4_
Sensitivity to inhibitor	Aminiguanidine, L-NNA, L-NAME, L-NMMA
Peroxisomal targeting signal (PTS)	Type 2 (PTS2)
Dependence of peroxisomal protein import system	PEX5, PEX7, PEX12, PEX13, Ca^2+^, CaM
Localization	Matrix

BH_4_, tetrabiopterin; PEX, peroxin; L-NNA, L-NG-Nitroarginine; L-NAME, Nω-Nitro-L-arginine methyl ester hydrochloride; L-NMMA, N^G^-Monomethyl-L-arginine, monoacetate salt; CaM, calmodulin.

**Table 2:** Some proteins from higher plant peroxisomes that undergo nitric oxide (NO)-derived posttranslational modifications (PTMs), either by *S*-nitrosation or tyrosine nitration.

Peroxisomal Enzyme	NO-Derived PTM	References
3-ketoacyl-CoA thiolase 1	*S*-nitrosation	[52]
Hydroxypyruvate reductase	*S*-nitrosation/nitration	[54,55,56]
Glycolate oxidase	*S*-nitrosation/nitration	[55,57,58]
Malate dehydrogenase	*S*-nitrosation/nitration	[55,59]
Catalase	*S*-nitrosation/nitration	[55,56,60,61]
CuZn superoxide dismutase (CSD3)	Nitration	[62]
Monodehydroascorbate reductase	*S*-nitrosation/nitration	[63]
